# Predicting P-Glycoprotein-Mediated Drug Transport Based On Support Vector Machine and Three-Dimensional Crystal Structure of P-glycoprotein

**DOI:** 10.1371/journal.pone.0025815

**Published:** 2011-10-04

**Authors:** Zsolt Bikadi, Istvan Hazai, David Malik, Katalin Jemnitz, Zsuzsa Veres, Peter Hari, Zhanglin Ni, Tip W. Loo, David M. Clarke, Eszter Hazai, Qingcheng Mao

**Affiliations:** 1 Virtua Drug Ltd., Budapest, Hungary; 2 Chemical Research Center, Hungary Academy of Sciences, Budapest, Hungary; 3 Delta Services Ltd., Budapest, Hungary; 4 Department of Medicine, Department and Biochemistry, University of Toronto, Toronto, Ontario, Canada; 5 Department of Pharmaceutics, School of Pharmacy, University of Washington, Seattle, Washington, United States of America; University of Cambridge, United Kingdom

## Abstract

Human P-glycoprotein (P-gp) is an ATP-binding cassette multidrug transporter that confers resistance to a wide range of chemotherapeutic agents in cancer cells by active efflux of the drugs from cells. P-gp also plays a key role in limiting oral absorption and brain penetration and in facilitating biliary and renal elimination of structurally diverse drugs. Thus, identification of drugs or new molecular entities to be P-gp substrates is of vital importance for predicting the pharmacokinetics, efficacy, safety, or tissue levels of drugs or drug candidates. At present, publicly available, reliable *in silico* models predicting P-gp substrates are scarce. In this study, a support vector machine (SVM) method was developed to predict P-gp substrates and P-gp-substrate interactions, based on a training data set of 197 known P-gp substrates and non-substrates collected from the literature. We showed that the SVM method had a prediction accuracy of approximately 80% on an independent external validation data set of 32 compounds. A homology model of human P-gp based on the X-ray structure of mouse P-gp as a template has been constructed. We showed that molecular docking to the P-gp structures successfully predicted the geometry of P-gp-ligand complexes. Our SVM prediction and the molecular docking methods have been integrated into a free web server (http://pgp.althotas.com), which allows the users to predict whether a given compound is a P-gp substrate and how it binds to and interacts with P-gp. Utilization of such a web server may prove valuable for both rational drug design and screening.

## Introduction

Human P-glycoprotein (P-gp, gene symbol *ABCB1*) is a large polytopic membrane protein belonging to the ATP-binding cassette (ABC) multidrug transporter superfamily. P-gp mediates efflux of a wide range of xenobiotics and endogenous compounds out of cells utilizing ATP hydrolysis as the source of energy for substrate translocation [1], [2], [3]. P-gp substrates are mainly hydrophobic and weakly amphipathic substances, including antibiotics, steroid hormones, chemotherapeutics, immunosuppressants, and anti-HIV protease inhibitors [3], [4], [5]. In addition to its ability of conferring multidrug resistance in cancer cells, P-gp is highly expressed in normal organs important for the absorption (the small intestine), elimination (the liver and kidney) and distribution (e.g., the placental and blood-brain barriers) of drugs and xenobiotics, and has been recognized as one of the most important drug transporters that are involved in clinically relevant drug disposition and drug-drug interactions [6]. P-gp consists of 1280 amino acid residues arranged into two homologous and symmetrical halves, each comprising one membrane-spanning domain (MSD) with six transmembrane (TM) α-helices followed by one cytosolic nucleotide-binding domain (NBD) [1], [3].

Since P-gp can influence the pharmacokinetics, efficacy, safety, and tissue levels of substrate drugs, increasing efforts are being devoted to investigating whether new molecular entities (NMEs) are potential P-gp substrates in early drug discovery and development. It is also very important to know if any existing drugs are P-gp substrates so that clinically important drug disposition and drug-drug interactions may be predicted. A variety of *in vitro* assays, including drug-stimulated ATPase activity, rhoadmine 123 or calcein-AM cellular accumulation, cell-based bi-directional transwell transport, drug permeability, and radioactive ligand binding have been used to classify drugs or drug candidates as P-gp substrates or non-substrates [7]. The data obtained from such *in vitro* studies can then be validated *in vivo* in preclinical animal models or in human subjects to assess the interactions of drugs or drug candidates with P-gp [7], [8], [9], [10]. Although the *in vitro* assays are highly efficient compared to *in vivo* studies, they are nonetheless still time-consuming, particularly when screening of a large number of NMEs is required in the early drug discovery stage. Therefore, *in silico* methods for predicting P-gp substrates and interactions are of high value for both rational drug discovery and screening. The availability of a vast amount of experimental transport data and the recently resolved X-ray structure of mouse P-gp [11] would now make it possible to develop much improved *in silico* prediction models.

Ligand-based and protein structure-based prediction methods are the two main classes of *in silico* prediction methods for protein-ligand interactions. Protein structure-based methods such as molecular docking allow prediction of protein-ligand interactions in atomic details, when high resolution experimental protein structures are available. Low resolution structures and homology models decrease the accuracy of docking calculations mostly due to the uncertainty of side chain conformations. However, a drawback of this method lies in the generation of a large number of potentially false positive results – that is, non substrates could also be calculated to bind to protein with high affinity. Thus, docking calculations alone cannot accurately predict P-gp substrates. On the other hand, ligand-based models, such as QSAR and SVM may be capable of predicting transport properties of test compounds based on their similarity to chemical structures of known substrates as well as their physicochemical properties. However, ligand-based methods do not provide information on protein-ligand interactions at the molecular level. Although a number of classification methodologies have been used in the development of QSAR models for P-gp substrates, there is no general rule concerning the selection of the best method for a specific classification problem. Penzotti et al. reported a computational ensemble pharmacophore model that had an overall classification rate of 80% for the training set and a prediction accuracy of 63% for a hold-out set [12]. Chang et al. applied pharmacophore models combined with screening of databases to retrieve molecules that bind to P-gp [13]. De Cerqueira Lima et al. developed a QSAR model for classification of drugs as P-gp substrates or non-substrates using a combination of methods and descriptor types [14]. Cabrera et al. used a topological substructural molecular design approach to predict whether a compound is a P-gp substrate and achieved a prediction accuracy of ∼71% on an external test set of marketed drugs [15]. Self-organizing maps (SOMs) represent another promising approach, and neural network can be used for classification purposes, too. Wang et al. [16] and Kaiser et al. [17] used SOMs to discriminate between P-gp inhibitors and substrates. In the latter study, the trained maps were subsequently used to identify highly active P-gp substrates in a virtual screening of a large compound library. Zhang et al. [18] applied the recursive partitioning method to classification of P-gp substrates and non-substrates based on *in vitro* bi-directional Caco-2 cell permeability and five descriptors of 14 marketed drugs and more than 100 discovery compounds. For a validation set of 46 compounds, the prediction accuracy was ∼72% and 89% for non-substrates and substrates, respectively [18].

Another ligand-based approach, namely, the support vector machine (SVM) technique, has been successfully used in a wide range of applications in computational biology [19]. The theory of SVM has been extensively reviewed elsewhere [20] and will only be briefly discussed here. The key point of SVM is to treat the objects that are to be classified as points in a high-dimensional space and to find a line (hyperplane) that separates them. Molecules are presented in the space with the help of molecular descriptors. The margin of the hyperplane is defined as the distance from the separating hyperplane to the nearest data point and SVM finds the maximum margin separating the hyperplane. The selection of this hyperplane maximizes the capability of SVM to predict the correct classification of new compounds. There are other hyperplane-based classification methods; however, SVM is distinct from them in the way how the hyperplane is selected. SVM is a mathematical entity, an algorithm used for maximizing a mathematical function with respect to collection of data. Since the SVM method is an excellent tool particularly for classification problems in chemometrics [21], it has been used to classify molecules as substrates or non-substrates of enzymes. For example, Mishra et al. developed an SVM-based web server for predicting the metabolizing capability of major isoforms of cytochrome P450 enzymes [22]. Likewise, SVM has also been used to predict P-gp substrates. Xue et al. used SVM and reported a prediction accuracy of 81% for P-gp substrates and 79% for non-substrates [23]. Huang et al. applied SVM optimized by a particle swarm and reported a prediction accuracy of 90% for P-gp substrates [24]. Most recently, Wang et al. developed several models using SVM based on a large training set of 212 compounds (131 P-gp substrates and 81 non-substrates), and the best model gave a prediction accuracy of 88% for a test set of 120 compounds [25].

It is important to note that the current *in silico* models are not readily available to experimental scientists, and hence do not significantly aid in the design of experiments for scientists who do not have access to these models. The goal of this study was to develop a predictive model and a free web server that can be used for *in silico* prediction of binding and transport characteristics of P-gp substrates for the scientific community. We have recently developed a similar system for evaluation of drug-human serum albumin binding interactions [26]. As discussed above, ligand-based and protein structure-based prediction methods are complementary to each other – that is, ligand-based methods may give a high prediction accuracy for given classes of drugs or drug candidates, while molecular docking calculations provide atomic details on protein-ligand interactions. Therefore, in the present study, both ligand-based (SVM) and P-gp structure-based (molecular docking) *in silico* methods were used for predicting P-gp-mediated transport and complex geometry. These prediction methods have now been integrated into a free web server (http://pgp.althotas.com). This web-based platform enables the users to predict the capability of P-gp to transport the query ligands and the complex geometries in the inward-facing conformation of human and mouse P-gp calculated using molecular docking tools.

## Methods

### Data Set

P-gp substrates and non-substrates used in this study were primarily taken from four compilations previously published [10], [27], [28], [29], which contain structurally diverse compounds. A number of compounds from other sources were also included in order to further increase molecular diversity of the data set. It should be noted that a number of contradictions exist in the literature in classification of compounds as P-gp substrates or non-substrates, which are discussed in details in the Results and Discussion section. Finally, 197 compounds (99 P-gp substrates and 98 non-substrates) were selected in our data set. 32 compounds were defined as “the external validation set” containing 16 P-gp substrates and 16 non-substrates and was set up for evaluation of prediction power with no bias as follows. Each compound was placed into n-dimensional space defined by the calculated molecular descriptors (see below). Correlation of the descriptors was used to calculate molecular similarity. The molecular space of P-gp substrates or non-substrates was then divided into 16 subsets and one molecule was randomly selected from each of these subsets to form the external validation set. All the compounds in the data sets are listed in Table S1.

### Support Vector Machine (SVM)

Structures of all P-gp substrates or non-substrates in the data sets were downloaded from the PubChem Database (http://pubchem.ncbi.nlm.nih.gov). All molecules were subjected to geometry optimization using the Molconvert software (ChemAxon, Budapest, Hungary), which applies the Dreiding molecular mechanics force field, and to calculation of the Gasteiger partial charges [30]. The DragonX software (www.talete.mi.it) was used to calculate a total of 3250 molecular descriptors for each molecule. The descriptors with >80% zero values and too small standard deviation values (less than 3%) were eliminated. The Libsvm software (www.csie.ntu.edu.tw/~cjlin/libsvm) was then used for SVM calculations. Linear, polynomial, and radial basis function (RBF) kernels were tested in the course of the study. The average Matthews coefficient of the external set based on 100 independent SVM calculations was 0.53, 0.18 or 0.54 using linear, polynomial or Gaussian RBF kernel, respectively. Therefore, in our SVM calculations, a Gaussian RBF was chosen as the kernel function:
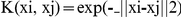
where γ is a kernel width parameter, x_i_ and x_j_ are instance label pairs, and K is the kernel function. In the training process, the regularization parameter ‘C’ and the kernel width parameter ‘γ’ were optimized using a grid search approach. The prediction power of SVM is greatly influenced by the selection of kernel and the parameters C and γ. The best combination of C and γ was selected by a grid-search with exponentially growing sequences of C and γ. Each combination of parameter choices was checked using cross validation, and the parameters with best cross-validation accuracy were selected. After the best parameters C and γ were found, the whole training set was trained again to generate the final model. The feature selection tool fselect.py (http://www.csie.ntu.edu.tw/~cjlin/libsvmtools) provided by the Libsvm developer was used to measure the relative importance of each feature. For each feature, an F-score can be calculated using fselect.py. Generally, the larger the F-score, the more likely the feature is discriminative. Therefore, this score was used as a feature selection criterion. Features with high F-scores were selected and then SVM was applied. High-F-score features were gradually added until the validation accuracy decreased. Descriptors were checked for their correlation. Among the descriptors with a correlation of >0.9, the descriptors with higher F-scores were kept for further SVM calculations. One hundred SVM calculations were run using the training data set, trained, and validated by cross-validation in such a way that a test set with a data size comparable to that of the external validation set was generated (i.e. a ratio of the training set to the test set of 0.8 was chosen, generating test sets of 33 compounds).

Prediction power of the above SVM method was evaluated based on the number of true positive (TP), true negative (TN), false positive (FP), and false negative (FN) predictions. Additional widely used parameters, namely accuracy (ACC), sensitivity (SE), specificity (SP) as well as the Matthews correlation coefficient (MCC) were also calculated using the equations given below [31].
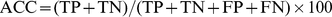


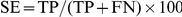


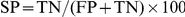


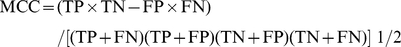



Prediction power is a measure of true hits in the entire calculations with both P-gp substrates and non-substrates included, whereas sensitivity and specificity reflect the prediction accuracies for P-gp substrates and non-substrates, respectively. The Matthews correlation coefficient considers over and under prediction and often provides a much more balanced evaluation of prediction than, for example, accuracy. MCC = 1 means a perfect prediction, whereas MCC = 0 indicates a random prediction.

### Homology modeling

The primary sequence of human P-gp was taken from Universal Protein Resource (http://www.uniprot.org) with the accession code P08183. For homology modeling of human P-gp, the X-ray structure of mouse P-gp (PDB code 3G60) was used as a template. The X-ray structure of the template was downloaded from the Protein Data Bank (http://www.rcsb.org). A sequence alignment between mouse and human P-gp was performed using ClustalW [32]. The alignment – as mouse and human P-gp share 87% sequence identity – was the same as that in previously published models [33]. Three-dimensional (3D) atomic models comprising all non-hydrogen atoms were generated by the Modeller9.8 package [34] using the refine.very slow option for simulated annealing. A bundle of ten models from random generation of the starting structures were calculated. The model possessing the lowest DOPE score was chosen for docking calculations. The quality of the model was evaluated using Procheck [35]. Ramachandran plot showed that 97% of the residues fell into the allowed region. The backbone root mean square deviation (RMSD) between the mouse P-gp template and the human P-gp model was 0.63 Å.

### Molecular docking

Molecular docking calculations were carried out using the Autodock Vina software [36] integrated in the Molecular Docking Server (http://www.dockingserver.com) [37]. Structures of the query ligands were optimized using the Dreiding force field integrated in the Molconvert program of ChemAxon (ChemAxon, Budapest, Hungary). Gasteiger partial charges were calculated on ligand atoms [30]. The X-ray structure of mouse P-gp (PDB code 3G60) and our human P-gp model were used for docking calculations. Polar hydrogen atoms were added to P-gp and Gasteiger partial charges were calculated using Autodock Tools. Water molecules and heteroatoms were removed from the structures, since water is implicitly included in Autodock. Simulation boxes were centered on ligands in the structures of P-gp-ligand complexes. A simulation box of 22×22×22 Å was used in each docking calculation with an exhaustiveness option of 8 (average accuracy).

### The web server

A free web server (http://pgp.althotas.com) has been developed based on the SVM and molecular docking methods described above. This web server allows the users to predict whether a query ligand may be a P-gp substrate, its binding property, and the geometry of P-gp-ligand complex. The Autodock Vina software was integrated in the web server for complex geometry calculations. The server was developed with PHP-MySQL and several external programs. The chemical structure of a query ligand can be uploaded or drawn in by the users using the built-in Chemaxon Marvin Java applet. The web server is linked to Pubchem so that ligands can be directly retrieved with text search. Structural conversions and 3D geometry optimization by the Dreiding method are carried out using the Molconvert software. 2-dimensional and 3-dimensional molecular descriptors are calculated using the DragonX software. The built-in SVM model of this study is used to predict P-gp substrates. The geometry of P-gp-ligand complex is predicted by docking calculations using the Autodock Vina algorithm [36] integrated in the web server. The X-ray structures of mouse P-gp (PDB codes 3G60 and 3G61) and the homology model of human P-gp are integrated in the web server.

## Results and Discussion

### SVM Calculations

SVM has some advantages – that is, it can treat both linear and non-linear data sets, and can be used for both classification and regression analysis; and the results can be easily interpreted. The method has gained popularity in a wide variety of biochemical applications, because it can be used for classification of small molecules on a given biological target [19]. Basically, compounds are represented with N calculated properties in the N dimensional space and the main task of an SVM calculation is to find a hyperplane in this space capable of separating the active molecules from the non-active ones. In order to build a reliable model, data selection is crucial in SVM. There are a number of experimental methods for determining P-gp substrate properties. The results of the different experimental methods might contradict. Stimulation of ATPase activity may be taken as an indication of direct interaction with P-gp. However, daunorubicin, which is an excellent P-gp substrate, does not significantly stimulate ATPase activity of P-gp, while verapamil and vincristine, also P-gp substrates, stimulate it well [10]. The MDCK cell monolayer model shows a net difference in substrate concentration between the apical and basal compartments only if the substrates are transported by P-gp at a rate that is higher than that of reuptake into cells (carrier mediated or passive diffusion). In direct drug binding studies, even if the binding affinity is high (low k_d_ values), the compounds may still not be well transported because the ratio between association (K_on_) and dissociation (K_off_) rates may be unfavorable. Moreover, different experimental conditions (e.g., pH and temperature) besides different methods used in determining substrate properties in different studies could also result in conflicting results for the same compounds. All the above factors often led to contradictions in the literature as to whether certain compounds are P-gp substrates or non-substrates. For example, yohimbine was considered as a P-gp substrate by Seelig [27], but was not classified as a P-gp substrate by Varma et al. [28]. Doxorubicin was considered as a non-substrate [10], [28], but was found to be transported by P-gp in other studies [38]. Similar situation exists for trazodone (non-substrate [10], [28] and substrate [16]) and venlaflaxine (non-susbtrate [39] and substrate [40]). Lidocaine, lovastatine, propranolol, and itraconazole were characterized to be non-substrates by Varma et al. [28] and Polli et al. [10], and simvastatine was characterized to be a non-substrate by Susanto et al. [41]; however, these compounds were all published as P-gp substrates or inhibitors in Pharmacology Weekly. Therefore, if the ability of compounds to be net-transported or have a high binding affinity, or the ability of compounds to stimulate ATPase activity is used synonymously to evaluate whether the compounds are P-gp substrates or non-substrates, this can lead to conflicting results on certain compounds and compromise correct classification in the training and validation data sets. Thus, in our data sets, a compound that had conflicting reports in the literature was treated as a P-gp substrate or non-substrate only if more independent studies confirmed its classification. We indicated the assay methods used to identify these compounds as P-gp substrates or non-substrates and references in Table S1.

SVM prediction of P-gp substrates or non-substrates was carried out by means of 100-fold cross-validation. Cross-validation serves two purposes: i) estimation of prediction power of the models generated, i.e. approximation of the general character of the models; and ii) comparison of prediction performance of the models and identification of the ‘best model’ for available data sets. The choice of the kernel is crucial in SVM calculations. Therefore, linear, polynomial, and Gaussian RBF kernels were tested in preliminary calculations. Our results showed that the average Matthews coefficient of the external set based on 100 independent SVM calculations (different ligand sets were used as the training and test data set in different runs) was 0.53, 0.18 or 0.54 using linear, polynomial or Gaussian RBF kernel, respectively. This is consistent with the results of previous P-gp SVM studies in which RBF was suggested as a reasonable choice [23], [24], [25]. Thus, the Gaussian RBF kernel was used throughout this study. The mean values of SVM prediction performance parameters of the 100 runs are presented in Table 1. These data indicate that the average accuracy of prediction for an external validation data set is near 80%. Note that specificity (SP) and sensitivity (SE) values are very close to each other (Table 1), indicating that the prediction for P-gp substrates and non-substrates is not discriminative, i.e. the model does not have preference for a random ligand to be a substrate or non-substrate. We did not compare our prediction results with those reported by others regarding prediction performance because such a direct comparison seems not appropriate due to large differences in compounds collected in data sets and data size. Nonetheless, Huang et al. [24] and Wang et al. [25] noticed that the prediction accuracy varied between 63 and 90% in 7 previous studies, and four of the studies gave a prediction accuracy of approximately 80%, which is comparable to that of this study.

**Table 1:** The mean values of SVM prediction performance parameters of 100 runs.

Data Set	ACC	SP	SE	MCC
Training	80	81	79	0.6064
Test	75	75	75	0.5117
External	76	77	74	0.5176

ACC, SP, SE, and MCC are accuracy, specificity, sensitivity, and the Matthews correlation coefficient, respectively.

**Figure 1:**
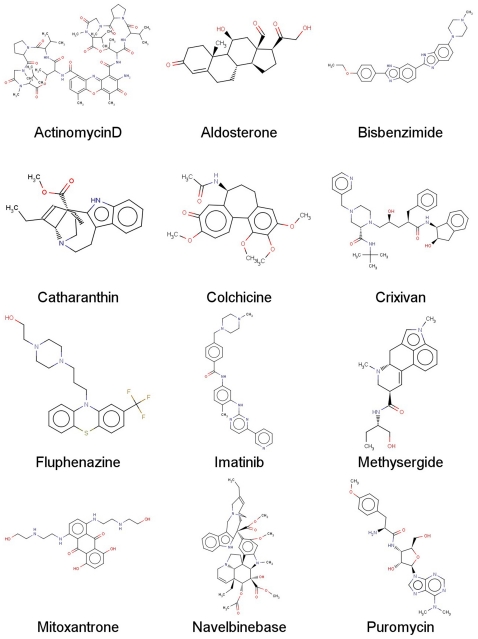
SVM prediction results for compounds in the external validation data set. Shown are chemical structures of the correctly predicted P-gp substrates by all the 6 best models.

**Figure 2:**
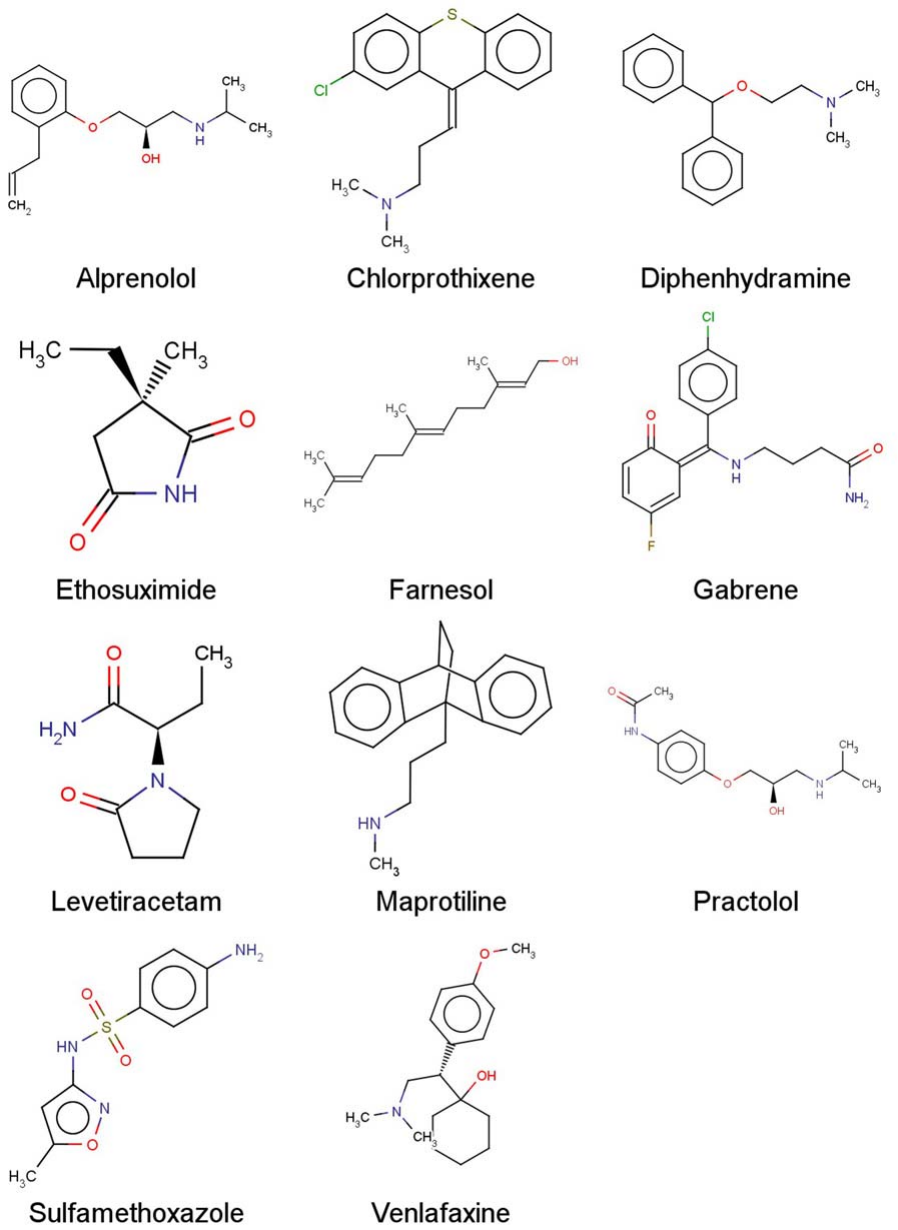
SVM prediction results for compounds in the external validation data set. Shown are chemical structures of the correctly predicted non-substrates of P-gp by all the 6 best models.

**Figure 3:**
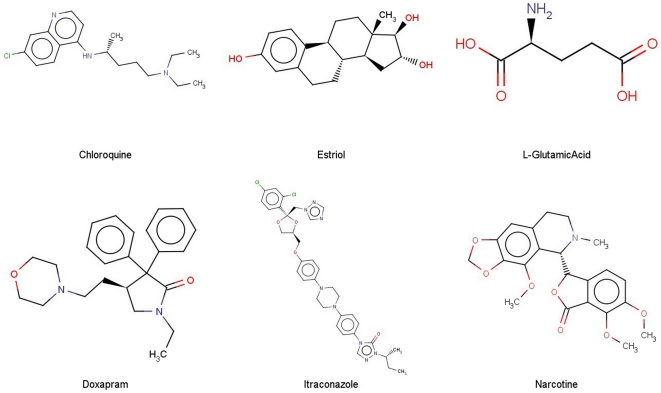
SVM prediction results for compounds in the external validation data set. Shown are chemical structures of the 6 incorrectly predicted P-gp substrates (top lane) and non-substrates (bottom lane) by all the 6 best models.

**Figure 4:**
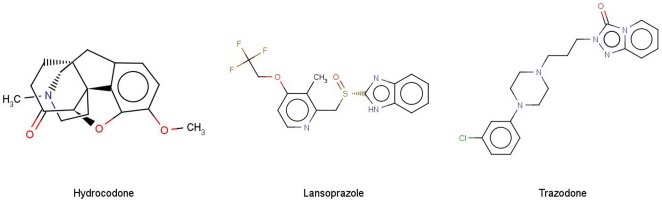
SVM prediction results for compounds in the external validation data set. Shown are chemical structures of the 3 ambiguously predicted P-gp substrates and non-substrates by different models.

There is no general rule concerning the selection of “the best model”. An obvious approach would be to select the model which provides the highest prediction accuracy for the training data set. However, this approach could be misleading because a model with the highest accuracy for a training data set does not necessarily give the highest accuracy for an independent external data set. For example, one of our models gave an accuracy of 94% for the training set and 75% for the test and external data sets. It is therefore necessary to take prediction accuracies for both the training and test sets into account when the “best model” is to be selected. Hence, the differences in prediction accuracies between the training and test data sets were calculated, and the models with the smallest differences (below 5%) were selected. We obtained 6 models using this criterion, and the prediction performance parameters of the 6 “best models” are summarized in Table S2. A large difference can be observed between specificity and sensitivity in the test set in models 1–4, indicating an overfitting of the models. These models contain a larger number of descriptors (48 descriptors in models 1–3 and 12 descriptors in model 4) compared to models 5 and 6 (6 descriptors). Indeed, it is known that the inclusion of too many descriptors in the SVM model decreases the accuracy of the performance for two reasons. First, the inclusion of too many descriptors may produce overfitted models. Second, the inclusion of unnecessary or irrelevant descriptors creates noise in the model. Thus, the model with the smallest number of descriptors and the highest MCC value for the external validation set was selected as the final model, which is the model 5 (Table S2). We noted that classification of individual compounds in the independent external data set as P-gp substrates or non-substrates were very similar in these models. The predictions of the external data set by the above-mentioned 6 best models are indicated in Table S3. Among the external data set of 32 molecules, twenty-three compounds were classified correctly by each model (Figures 1 and 2), and 6 compounds (chloroquine, estriol, L-glutamic acid, doxapram, itraconazole, and narcotine) were misclassified unambiguously by all the 6 models (Figure 3). We note that all the unambiguously misclassified substrates are relatively small in size, whereas the misclassified non-substrates possess larger molecular weights (Figure 3). Only 3 compounds (lansoprazole, hydrocodone, and trazodone) were not uniformly predicted as P-gp substrates or non-substrates by the 6 models (Figure 4).

The final model (the model 5) is based on the following descriptors: ATS1m (Broto-Moreau autocorrelation of a topological structure-lag 1/weighted by atomic masses), EEig12x (Eigenvalue 12 from edge adj. matrix weighted by edge degrees), ESpm02d (Spectral moment 02 from edge adj. matrix weighted by dipole moments), BELv6 (lowest eigenvalue n. 6 of Burden matrix/weighted by atomic van der Waals volumes), BELe6 (lowest eigenvalue n. 6 of Burden matrix/weighted by atomic Sanderson electronegativities), BELp6 (highest eigenvalue n. 6 of Burden matrix/weighted by atomic polarizabilities). These descriptors are well suited to quantify transport properties relevant to P-gp substrates. This is manifested by the fact that the descriptor values are weighted by the atomic mass (ATS1m), the size (BELv6), the polarizibility and logP (BELp6), electronegativity (BELe6), and dipole moment (ESpm02d) of the ligands of the data set. Our findings are consistent with previous SVM studies, namely, it has been showed that 60% of the molecular descriptors important for P-gp substrates are of topological nature [42]. The X-Ray structure of mouse P-gp reveals a large hydrophobic binding site packed with a number of aromatic residues. Thus, substrate binding seems to be a result of a combination of hydrophobic, aromatic, and electrostatic interactions. The presence of aromatic residues in the binding pocket explains the role of polarizability and logP in the descriptors.

### Molecular docking calculations

**Figure 5:**
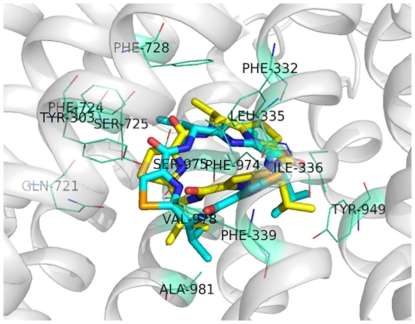
The Docking of QZ59-RRR to P-gp. Docking of QZ59-RRR to the X-ray structure of P-gp-QZ59-RRR complex (PDB code: 3G60) was performed using the Autodock Vina algorithm to validate the accuracy of docking calculations. The geometry of P-gp-QZ59-RRR complex obtained by docking calculations (yellow carbons) was compared with that of the P-gp-QZ59-RRR complex in the X-ray structure (blue carbons). Potential binding residues of P-gp for QZ59-RRR in the internal cavity are indicated.

Several studies have shown that the correlation between the experimental and calculated binding energies is below 0.6 with any docking software available on the market [43]. Therefore, docking itself is not reliable for differentiating compounds between substrates and non-substrates. Thus, we predicted P-gp substrates using the ligand-based SVM method as described above. However, since molecular docking calculations have been shown to yield accurate complex geometry predictions in a number of protein families [43], we used molecular docking to predict substrate-P-gp interactions at an atomic level. It should be noted that the P-gp structure - including the substrate translocation pore - is treated as a rigid object in the docking procedure, which is not the case experimentally. However, since the drug-binding conformation of P-gp (an inward-facing closed apo conformation) was used, the docking results do provide insights into the possible complex geometry of ligand-bound P-gp prior to ATP hydrolysis. To validate if docking can predict accurately the geometry of P-gp-ligand complex, the cyclic peptide P-gp inhibitor (QZ59-RRR) was re-docked to the original mouse P-gp structure with QZ59-RRR bound (PDB code 3G60), and the experimental and calculated complex geometries were compared. As shown in Figure 5, QZ59-RRR was docked to the experimentally determined structure of mouse P-gp with a high accuracy with a RMSD value of 1.27 Å, thus confirming the capability of docking calculations to predict P-gp-ligand complex geometry. Obviously, the side chains of P-gp residues are appropriately oriented for QZ59-RRR, which is not the case for other compounds. Therefore, docking calculations of other ligands are expected to yield lower prediction accuracies than this validation with QZ59-RRR.

We next investigated whether docking calculations to our homology model of human P-gp can predict complex geometry. In the modeling, mouse P-gp was used as a template. As human and mouse P-gp share 87% amino acid identity, a reliable homology model of human P-gp can be built with simple modeling procedure. This high sequence identity makes the sequence alignment obvious (data not shown). Indeed, recent studies have revealed identical sequence alignments in developing homology models of human P-gp [44]. The inward-facing closed apo conformation of P-gp has been used to predict residues that are experimentally implicated in drug transport and demonstrate that P-gp has a high affinity for drug substrates [45].

**Figure 6:**
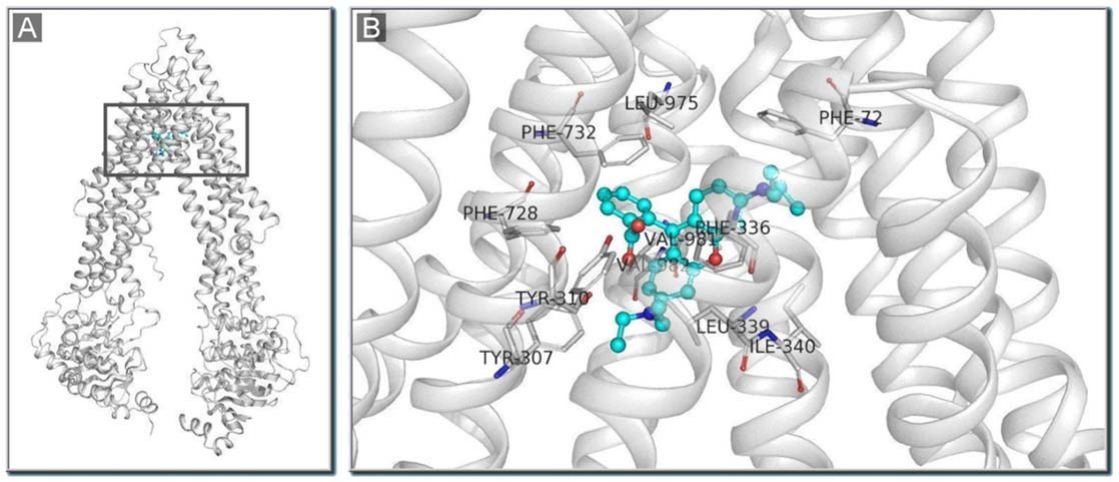
Location of rhodamine B in P-gp and the geometry of the P-gp-rhodamine B complex. Docking of rhodamine B to the X-ray structure of P-gp (PDB code: 3G60) was performed using the Autodock Vina software. **A**. Location of rhodamine B (blue carbons) in the internal cavity of the inward-facing from of P-gp. **B**. The geometry of the P-gp-rhodamine B (blue carbons) complex. Potential binding residues of P-gp for rhodamine B in the internal cavity are indicated.

To validate our homology model of human P-gp, rhodamine B, a known P-gp substrate with experimental binding data available was docked to our human P-gp model. Rhodamine B is a highly hydrophobic compound and a well-characterized P-gp substrate [46]. It was included in our SVM training set and was correctly predicted to be a P-gp substrate by our SVM model. Since numerous studies have shown that the drug-binding cavity in P-gp is primarily formed by TM helices [11], [47], we anticipate that rhodamine B would most likely bind to P-gp in the MSD through hydrophobic interactions. Indeed, such interactions and location of the rhodamine B binding site in P-gp can be directly visualized by docking calculations (Figure 6). Rhodamine B may interact with the following aromatic residues that form a binding pocket: Phe72, Phe336, Phe728, Phe732, Tyr307, and Tyr310. These residues may have hydrophobic interactions with the hydrophobic side chains of rhodamine B as well as pi-pi interactions with the aromatic ring system of the compound. Hydrophobic side chains of other residues, namely Leu975, Val981 and Val982, are also possibly within the interacting distance. Additionally, two cation-pi interactions were observed between the two positively charged nitrogens of rhodamine B and Phe728 and Tyr307. The carboxylate side chain of rhodamine B could form a double hydrogen bond with the hydroxyl groups of Tyr307 and Tyr310. These results of docking calculations appear to be consistent with experimental data. For example, activities of the human P-gp mutants, I340C (in TM6), L975C (in TM12), V981C (in TM12), and V982C (in TM12), were found to be highly protected from inhibition by MTS-rhodamine by pre-treatment with rhodamine B, indicating that these residues likely participate in rhodamine B binding to human P-gp [48]. It is important to note that several binding residues for rhodamine B identified in this study (e.g., Phe336/(Ile in mouse P-gp), Phe728, and Leu975 (Ser in mouse P-gp) were the same or proximate to the binding residues for ligands such as QZ59-RRR identified in the X-ray structure of mouse P-gp [11] which served as the template for the human P-gp model, suggesting that these hydrophobic ligands may occupy distinct, but overlapping binding sites or bind to different regions within the same binding site in the large drug-binding cavity.

### A free web server

To make the SVM and docking methods publicly available, we have developed a free web server (http://pgp.althotas.com) which enables the users to predict if a compound is a P-gp substrate, as well as its complex geometry in P-gp. The PubChem database is integrated so that any compounds can be searched and submitted by their names. With this web server, any molecule of interest can be searched by its name, uploaded in PDB, mol, mol2, hin, or SMILES format or drawn in using a Marvin applet by the users. After submitting the molecule, the web server performs: i) SVM prediction for P-gp substrate; ii) identification of physicochemical parameters of the ligand such as molecular weight, logP, and polarizability; and iii) docking calculations for predicting the complex geometry, docking energy, and interaction surface of the ligand in the structure of mouse P-gp and the homology model of human P-gp.

In summary, P-gp confers multidrug resistance in cancer cells [1], [3], and is also involved in clinically relevant drug disposition and drug-drug interactions [6]. Therefore, to evaluate the pharmacokinetics, safety, and efficacy of drugs or NMEs, it is of important value to predict whether they are P-gp substrates and how they might interact with P-gp, which, at present, remains largely unknown. As an effort to address this issue, in the present study, we have applied an SVM method to predict potential P-gp substrates based on a relatively large data set of 197 known P-gp substrates and non-substrates. This SVM method showed a prediction accuracy of ∼80% in an independent external data set of 32 compounds. The selected descriptors were related to molecular properties such as molecular weight, electronegativity and polarizability. Since the 3D crystal structures of mouse P-gp with or without ligands are already available [11], homology model of human P-gp was constructed and interactions of P-gp substrates with particular residues in the transporter could be investigated by molecular docking calculations. We have shown, by the example of rhodamine B, a known P-gp substrate, that molecular docking calculations can predict the complex geometry in an internal drug-binding cavity of P-gp that is consistent with experimental data. Therefore, SVM prediction and molecular docking calculations may prove valuable for prediction of P-gp substrates as well as analysis of P-gp-substrate interactions at the molecular level, and hence facilitate both rational drug design and screening. This approach is particularly useful and cost-effective in the early drug discovery stage. To make the prediction methods described in this study available to the large scientific community, a free web server (http://pgp.althotas.com) has been developed which integrates both the SVM prediction and molecular docking calculations. To the best of our knowledge, this is the first free web server for predicting P-gp-mediated drug transport and complex geometry.

## Supporting Information

Table S1 **P-gp substrates (class 1) and non-substrates (class 0) in the training or test (t) and the independent external validation (e) data sets used for SVM prediction.**
(DOCX)

Table S2 **SVM prediction performance parameters of the 6 best models.**
(DOCX)

Table S3 **Prediction results in the external validation data set by the 6 best models.**
(DOCX)
