# Supplementary material for: Predicting P-Glycoprotein-Mediated Drug Transport Based On Support Vector Machine and Three-Dimensional Crystal Structure of P-glycoprotein
Source: PLoS One. 2011 Oct 4;6(10):e25815. doi: 10.1371/journal.pone.0025815 (PMC3186768; doi:10.1371/journal.pone.0025815)
Supplement: Table S1 — P-gp substrates (class 1) and non-substrates (class 0) in the training or test (t) and the independent external validation (e) data sets used for SVM prediction. (DOCX) [file pone.0025815.s001.docx]

**Table S1. P-gp substrates (class 1) and non-substrates (class 0) in the training or test (t) and the independent external validation (e) data sets used for SVM prediction**.

| Compound | DrugBank/Pubchem ID | Set | Class | Assay | Reference |
| --- | --- | --- | --- | --- | --- |
| Acetyldigitoxin | DB00511 | t | 1 | Permeability in Caco-2 cells | [1] |
| Acrivastine | CID_5284514 | t | 1 | ATPase, Calcein AM accumulation, permeability | [2] |
| Amiodarone | CID_2157 | t | 1 | Drug efflux meacurements | [3] |
| Amprenavir | CID_65016 | t | 1 | ATPase, Calcein AM accumulation, permeability | [2] |
| Astemizole | CID_2247 | t | 1 | ATPase, Rhodamine123 and Calcein AM accumulation | [4] |
| Atorvastatin | CID_60823 | t | 1 | Bi-directional transwell transport with LLC-PK1/MDR1 cells | [5] |
| Azidopine | CID_656406 | t | 1 | Drug displacement study | [6] |
| Bepredil | CID_2351 | t | 1 | Drug binding assay | [7] |
| Betamethasone | DB00443 | t | 1 | Bi-directional transwell transport with LLC-PK1/MDR1 cells | [8] |
| Bisantrene | CID_5351322 | t | 1 | ATPase, drug binding assay | [9] |
| Brompheniramine | DB00835 | t | 1 | Permeability with MDCK/MDR1 cells | [10] |
| Cefazolin | CID_33255 | t | 1 | Drug binding assay, accumulation studies, MES-SA cells | [11] |
| Cefoperazone | CID_44187 | t | 1 | Drug binding assay, accumulation studies with MES-SA cells | [11] |
| Cepharanthine | CID_10206 | t | 1 | Drug displacement studies | [12] |
| Cerivastatin | DB00439 | t | 1 | Bi-directional transwell transport with Caco-2 and L-MDR1 cells | [13] |
| Cetirizine | CID_2678 | t | 1 | Calcein AM accumulation, permeability | [14] |
| Cinchonidine | CID_101744 | t | 1 | Drug efflux | [3] |
| Citalopram | DB00215 | t | 1 | Bi-directional transwell transport with MDCKII, MDCKII/MDR1, MDCKII/Mdr1a cells, ATPase, and Calcein AM accumulation inhibition | [15] |
| Cortisone acetate | DB01380 | t | 1 | Bi-directional transwell transport with LLC-PK1/MDR1 cells | [8] |
| Cyclosporin A | CID_5284373 | t | 1 | ATPase, Rhodamine123 and Calcein AM accumulation | [16] |
| Daunorubicin | CID_30323 | t | 1 | Drug displacement study | [17] |
| Dexamethasone | CID_5743 | t | 1 | Bi-directional transwell transport with LLC-PK1/MDR1 cells | [18] |
| Dibucaine | CID_3025 | t | 1 | Drug displacement study | [19] |
| Dicloxacillin | DB00485 | t | 1 | Bi-directional transwell transport with MDCKII and MDCKII/MDR1 cells | [20] |
| Digitoxin | DB01396 | t | 1 | Permeability with Caco-2 cells | [1] |
| Digoxin | CID_2724385 | t | 1 | Bi-directional transwell transport with LLC-PK1/MDR1 cells | [18] |
| Diltiazem | CID_39186 | t | 1 | Drug displacement study | [19] |
| Dipyridamole | CID_3108 | t | 1 | Drug displacement study | [21] |
| Domperidone | CID_3151 | t | 1 | Bi-directional transwell transport with LLC-PK1/MDR1 cells | [18] |
| Elancoban | CID_28263 | t | 1 | ATPase, Calcein AM accumulation, permeability | [2] |
| Eletriptan | CID_77993 | t | 1 | Bi-directional transwell transport with LLC-PK1/MDR1 cells | [22] |
| Emetine | CID_10219 | t | 1 | Drug efflux measurements | [23] |
| Erythromycin | DB00199 | t | 1 | ATPase, Rhodamine123 and Calcein AM accumulation | [4] |
| Estrone | DB00655 | t | 1 | Bi-directional transwell transport with MDCKII and MDCKII/MDR1 cells, ATPase | [24] |
| Etoposide | CID_36462 | t | 1 | ATPase, Rhodamine123 and Calcein AM accumulation | [4] |
| Famciclovir | CID_3324 | t | 1 | Calcein AM accumulation, permeability | [14] |
| Fexofenadine | DB00950 | t | 1 | Permeability with MDCKII/MDR1 cells | [10] |
| Flunisolide | DB00180 | t | 1 | Bi-directional transwell transport with Calu-3 cells | [25] |
| Flupentixol | CID_5281881 | t | 1 | Drug displacement study | [26] |
| Gallopamil | CID_1234 | t | 1 | Drug displacement study | [21] |
| Hydrocortisone | CID_5754 | t | 1 | Bi-directional transwell transport with LLC-PK1/MDR1 cells | [27] |
| Labetalol | CID_3869 | t | 1 | Calcein AM accumulation, permeability | [14] |
| Loperamide | CID_3955 | t | 1 | Bi-directional transwell transport with LLC-PK1/MDR1 cells | [18] |
| Mequitazine | CID_4066 | t | 1 | Calcein AM accumulation, permeability | [14] |
| Methylprednisolone | DB00959 | t | 1 | Bi-directional transwell transport with LLC-PK1/MDR1 cells | [8] |
| Mibefradil | DB01388 | t | 1 | Calcein AM accumulation, ATPase, transcellular transport | [4] |
| Morphine 6-glucuronide | CID_5360621 | t | 1 | Drug displacement study | [28] |
| Nalbuphine | CID_5311304 | t | 1 | Calcein AM accumulation, permeability | [14] |
| Nelfinavir | CID_64143 | t | 1 | ATPase, Calcein AM accumulation, permeability | [2] |
| Neostigmine | CID_4456 | t | 1 | ATPase, Calcein AM accumulation, permeability | [2] |
| Omeprazole | DB00338 | t | 1 | Bi-directional transwell transport with Caco-2 and L-MDR1 cells | [29] |
| Ondansetron | CID_4595 | t | 1 | Bi-directional transwell transport with LLC-PK1/MDR1 cells | [18] |
| Oseltamivir | DB00198 | t | 1 | Bi-directional transwell transport across with LLC-GA5-COL150 cells | [30] |
| Paclitaxel | CID_36314 | t | 1 | ATPase, Calcein AM accumulation, permeability | [2] |
| Pantoprazole | DB00213 | t | 1 | Bi-directional transwell transport with L-MDR1 and Caco-2 cells | [29] |
| Phenoxazine | CID_67278 | t | 1 | Drug displacement study | [31] |
| Phenytoin | CID_1775 | t | 1 | Bi-directional transwell transport with LLC-PK1/MDR1 cells | [18] |
| Phosphatidylserine | DB00144 | t | 1 | Bi-directional transwell transport with EPG85-257P cells | [32] |
| Pirenzepine | CID_4848 | t | 1 | Calcein AM accumulation, permeability | [14] |
| Pravastatin | DB00175 | t | 1 | Drug displacement study | [33] |
| Prazosin | CID_4893 | t | 1 | Efflux transport measurements, Calcain AM accumulation, and ATPase | [34] |
| Prednisolone | DB00860 | t | 1 | Bi-directional transwell transport with LLC-PK1/MDR1 cells | [8] |
| Prednisone | DB00635 | t | 1 | Bi-directional transwell transport with LLC-PK1/MDR1 cells | [8] |
| Quinidine | CID_441074 | t | 1 | ATPase, Rhodamine123 and Calcein AM accumulation | [4] |
| Reserpine | CID_5770 | t | 1 | Drug displacement studies | [12] |
| Rhodamine 123 | CID_65218 | t | 1 | Photoaffinity labeling | [35] |
| Risperidone | CID_5073 | t | 1 | Calcein AM accumulation, permeability | [14] |
| Ritonavir | CID_392622 | t | 1 | ATPase, Calcein AM accumulation, permeability | [2] |
| Saquinavir | CID_441243 | t | 1 | ATPase, Calcein AM accumulation, permeability | [2] |
| SDB-ethylenediamine | CID_6438957 | t | 1 | Drug displacement studies | [12] |
| Sirolimus | CID_5284616 | t | 1 | Bi-directional transwell transport with CYP3A4 transfected Caco-2 cells | [36] |
| Spiperone | CID_5265 | t | 1 | Drug displacement study | [19] |
| Tacrolimus | CID_445643 | t | 1 | Drug displacement study | [37] |
| Terfenadine | DB00342 | t | 1 | ATPase, Rhodamine123 and Calcein AM accumulation | [17] |
| Topotecan | CID_60700 | t | 1 | Cytotoxicity assay | [38] |
| Tramadol | DB00193 | t | 1 | Bi-directional transwell transport with Caco-2 cells | [39] |
| Trifluoperazine | CID_5566 | t | 1 | Drug displacement study | [40] |
| Triflupromazine | CID_5568 | t | 1 | Drug displacement study | [40] |
| Trimethoprim | CID_5578 | t | 1 | Bi-directional transwell transport with MDCKII and MDCKII/MDR1 cells | [20] |
| Vinblastine | DB00570 | t | 1 | ATPase, Rhodamine123 and Calcein AM accumulation | [4] |
| Vincristine | DB00541 | t | 1 | Drug displacement study | [41] |
| Vindoline | CID_16596 | t | 1 | Drug displacement study | [17] |
| Zolmitriptan | CID_60857 | t | 1 | Calcein AM accumulation, permeability | [14] |
| Amantadine | CID_2130 | t | 0 | ATPase, Calcein AM accumulation, permeability | [2] |
| Amitriptyline | CID_2160 | t | 0 | Calcein AM accumulation, permeability | [14] |
| Antipyrine | CID_2206 | t | 0 | Calcein AM accumulation, permeability | [14] |
| Atenolol | CID_2249 | t | 0 | Calcein AM accumulation, permeability | [14] |
| Biperiden | CID_2381 | t | 0 | Calcein AM accumulation, permeability | [14] |
| Bromocriptine | CID_31101 | t | 0 | Calcein AM accumulation, permeability | [14] |
| Bufuralol | CID_71733 | t | 0 | Calcein AM accumulation, permeability | [14] |
| Buspirone | CID_2477 | t | 0 | Calcein AM accumulation, permeability | [14] |
| Caffeine | DB00201 | t | 0 | Bi-directional transwell transport with MDCKII, MDCKII/MDR1, and MDCKII/Mdr1a cells, ATPase, and Calcein AM accumulation inhibition | [15] |
| Carbamazepine | CID_2554 | t | 0 | Bi-directional transwell transport with MDCKII/MDR1 cells | [42] |
| Carisoprodol | DB00395 | t | 0 | Bi-directional transwell transport with MDCKII, MDCKII/MDR1, and MDCKII/Mdr1a cells, ATPase, and Calcein AM accumulation inhibition | [15] |
| Chlorpromazine | DB00477 | t | 0 | Bi-directional transwell transport with MDCKII, MDCKII/MDR1, and MDCKII/Mdr1a cells, ATPase, and Calcein AM accumulation inhibition | [15] |
| Clomipramine | CID_2801 | t | 0 | Calcein AM accumulation, permeability | [14] |
| Clonidine | CID_2803 | t | 0 | Calcein AM accumulation, permeability | [14] |
| Cyclobenzaprine | CID_2895 | t | 0 | Calcein AM accumulation, permeability | [14] |
| Desipramine | DB01151 | t | 0 | Calcein AM accumulation, permeability | [14] |
| Diazepam | DB00829 | t | 0 | Bi-directional transwell transport with MDCKII, MDCKII/MDR1, and MDCKII/Mdr1a cells, ATPase, and Calcein AM accumulation inhibition | [15] |
| D-mannitol | CID_6251 | t | 0 | ATPase, Calcein AM accumulation, permeability | [2] |
| Doxepine | CID_667477 | t | 0 | Knockout mice | [43] |
| Doxorubicin | DB00997 | t | 0 | ATPase, Calcein AM accumulation, permeability | [2] |
| Doxylamine | CID_3162 | t | 0 | Calcein AM accumulation, permeability | [14] |
| Epinephrine | CID_5816 | t | 0 | Drug displacement study | [44] |
| Flumazenil | CID_3373 | t | 0 | Calcein AM accumulation, permeability | [14] |
| Fluoxetine | DB00472 | t | 0 | Calcein AM accumulation, permeability | [14] |
| Flurazepam | CID_3393 | t | 0 | Calcein AM accumulation, permeability | [14] |
| Fluvoxamine | DB00176 | t | 0 | Calcein AM accumulation, permeability | [14] |
| Guanfacine | CID_3519 | t | 0 | Calcein AM accumulation, permeability | [14] |
| Haloperidol | CID_3559 | t | 0 | Calcein AM accumulation, permeability | [14] |
| Hydroxyzine | DB00557 | t | 0 | Drug displacement study | [45] |
| Imipramine | CID_3696 | t | 0 | Calcein AM accumulation, permeability | [14] |
| Indomethacin | CID_3715 | t | 0 | Calcein AM accumulation, permeability | [14] |
| Ketamine | CID_3821 | t | 0 | Calcein AM accumulation, permeability | [14] |
| Ketoconazole | DB01026 | t | 0 | ATPase, Calcein AM accumulation, permeability | [2] |
| Lamotrigine | DB00555 | t | 0 | Bi-directional transwell transport with MDCKII, MDCKII/MDR1, and MDCKII/Mdr1a cells, ATPase, and Calcein AM accumulation inhibition | [15] |
| Lidocaine | DB00281 | t | 0 | ATPase, Calcein AM accumulation, permeability | [2] |
| Lorcainide | CID_42884 | t | 0 | Calcein AM accumulation, permeability | [14] |
| Lovastatin | DB00227 | t | 0 | Bi-directional transwell transport with LLC-PK1/MDR1, L-MDR1 and Caco-2 cells | [46] |
| Mebendazole | DB00643 | t | 0 | ATPase, Calcein AM accumulation, permeability | [2] |
| Mecysteine | CID_29145 | t | 0 | ATPase, drug binding assay | [9] |
| Mephentermrine | CID_3677 | t | 0 | Calcein AM accumulation, permeability | [14] |
| Meprobamate | CID_4064 | t | 0 | Calcein AM accumulation, permeability | [14] |
| Metergoline | CID_28693 | t | 0 | Calcein AM accumulation, permeability | [14] |
| Methotrexate | CID_126941 | t | 0 | ATPase, Calcein AM accumulation, permeability | [2] |
| Methylphenidate | DB00422 | t | 0 | Bi-directional transwell transport with MDCKII, MDCKII/MDR1, and MDCKII/Mdr1a cells, ATPase, and Calcein AM accumulation inhibition | [15] |
| Metoclopramide | DB01233 | t | 0 | Bi-directional transwell transport with MDCKII, MDCKII/MDR1, and MDCKII/Mdr1a cells, ATPase, and Calcein AM accumulation inhibition | [15] |
| Metoprolol | CID_4171 | t | 0 | Calcein AM accumulation, permeability | [14] |
| Midazolam | DB00683 | t | 0 | Bi-directional transwell transport with MDCKII, MDCKII/MDR1, and MDCKII/Mdr1a cells, ATPase, and Calcein AM accumulation inhibition | [15] |
| Naloxone | CID_5284596 | t | 0 | Calcein AM accumulation, permeability | [14] |
| Naltrexone | DB00704 | t | 0 | Calcein AM accumulation, permeability | [14] |
| Nordazepam | CID_2997 | t | 0 | Calcein AM accumulation, permeability | [14] |
| Norethindrone | DB00717 | t | 0 | Bi-directional transwell transport with MDCKII/MDR1 cells, ATPase | [24] |
| Nortriptyline | DB00540 | t | 0 | Calcein AM accumulation, permeability | [14] |
| Ouabain | DB01092 | t | 0 | Permeability with Caco-2 cells | [1] |
| Oxprenolol | CID_4631 | t | 0 | Calcein AM accumulation, permeability | [14] |
| Paroxetine | DB00715 | t | 0 | Bi-directional transwell transport with MDCKII, MDCKII/MDR1, and MDCKII/Mdr1a cells, ATPase, and Calcein AM accumulation inhibition | [15] |
| Perphenazine | CID_4748 | t | 0 | Calcein AM accumulation, permeability | [14] |
| Pheniramine | CID_4761 | t | 0 | Calcein AM accumulation, permeability | [14] |
| Procyclidine | CID_4919 | t | 0 | Calcein AM accumulation, permeability | [14] |
| Promazine | CID_4926 | t | 0 | Calcein AM accumulation, permeability | [14] |
| Promethazine | DB01069 | t | 0 | Calcein AM accumulation, permeability | [14] |
| Propoxyphene | DB00647 | t | 0 | Bi-directional transwell transport with MDCKII, MDCKII/MDR1, and MDCKII/Mdr1a cells, ATPase, and Calcein AM accumulation inhibition | [15] |
| Propranolol | CID_4946 | t | 0 | ATPase, Calcein AM accumulation, permeability | [2] |
| Pyridostigmine | CID_4991 | t | 0 | ATPase, Calcein AM accumulation, permeability | [2] |
| Reserpic acid | CID_65747 | t | 0 | Drug displacement study | [47] |
| Scopolamine | CID_5184 | t | 0 | Calcein AM accumulation, permeability | [14] |
| Selegiline | CID_26757 | t | 0 | Calcein AM accumulation, permeability | [14] |
| Sertraline | DB01104 | t | 0 | Bi-directional transwell transport with MDCKII, MDCKII/MDR1, and MDCKII/Mdr1a cells, ATPase, and Calcein AM accumulation inhibition | [15] |
| S-farnesylcysteine | CID_6438372 | t | 0 | ATPase activity | [9] |
| Simvastatin | DB00641 | t | 0 | Bi-directional transwell transport with LLC-PK1/MDR1 cells | [5] |
| Sulfasalazine | DB00795 | t | 0 | Bi-directional transwell transport with Caco-2 cells | [48] |
| Sulpiride | DB00391 | t | 0 | Bi-directional transwell transport with MDCKII, MDCKII/MDR1, and MDCKII/Mdr1a cells, ATPase, and Calcein AM accumulation inhibition | [15] |
| Sumatriptan | CID_5358 | t | 0 | ATPase, Calcein AM accumulation, permeability | [2] |
| Tacrine | CID_1935 | t | 0 | Calcein AM accumulation, permeability | [14] |
| Testosterone | DB00624 | t | 0 | ATPase, Calcein AM accumulation, permeability | [2] |
| Thiopental | DB00599 | t | 0 | Bi-directional transwell transport with MDCKII, MDCKII/MDR1, and MDCKII/Mdr1a cells, ATPase, and Calcein AM accumulation inhibition | [15] |
| Triamterene | CID_5546 | t | 0 | ATPase, Calcein AM accumulation, permeability | [2] |
| Trimipramine | DB00726 | t | 0 | Calcein AM accumulation, permeability | [14] |
| Warfarin | CID_6691 | t | 0 | Calcein AM accumulation, permeability | [14] |
| Yohimbine | CID_8969 | t | 0 | ATPase, Calcein AM accumulation, permeability | [2] |
| Zolpidem | CID_5732 | t | 0 | Calcein AM accumulation, permeability | [14] |
| Actinomycin D | CID_2019 | e | 1 | Calcein AM accumulation, permeability | [14] |
| Aldosterone | CID_5839 | e | 1 | Bi-directional transwell transport with LLC-PK1/MDR1 cells | [27] |
| Catharanthin | CID_197771 | e | 1 | Drug dsplacement study | [17] |
| Chloroquine | CID_2719 | e | 1 | ATPase, Calcein AM accumulation, permeability | [2] |
| Colchicine | DB01394 | e | 1 | ATPase, Calcein AM accumulation, permeability | [2] |
| Estriol | DB04573 | e | 1 | Bi-directional transwell transport with MDCKII/MDR1 cells, ATPase activity | [24] |
| Fluphenazine | CID_3372 | e | 1 | Bi-directional transwell transport with MCF-7 cells | [40] |
| Hoechst33342 | CID_1464 | e | 1 | ATPase, Calcein AM accumulation, permeability | [2] |
| Imatinib | DB00619 | e | 1 | Cytotoxicity and transepithelial transport assays | [49] |
| Indinavir | CID_5362440 | e | 1 | ATPase, Calcein AM accumulation, permeability | [2] |
| Lansoprazole | DB00448 | e | 1 | Bi-directional transwell transport with Caco-2 and L-MDR1 cells | [29] |
| L-Glutamic Acid | DB00142 | e | 1 | Drug displacement study | [50] |
| Methysergide | CID_9681 | e | 1 | Calcein AM accumulation, permeability | [14] |
| Mitoxantrone | CID_4212 | e | 1 | ATPase, Calcein AM accumulation, permeability | [2] |
| Puromycin | DB08437 | e | 1 | ATPase, Calcein AM accumulation, permeability | [2] |
| Vinorelbine | CID_60780 | e | 1 | ATPase, Calcein AM accumulation, permeability | [2] |
| Alprenolol | CID_2119 | e | 0 | Calcein AM accumulation, permeability | [14] |
| Chlorprothixene | CID_667467 | e | 0 | Calcein AM accumulation, permeability | [14] |
| Diphenhydramine | CID_3100 | e | 0 | Calcein AM accumulation, permeability | [14] |
| Doxapram | CID_3156 | e | 0 | Calcein AM accumulation, permeability | [14] |
| Ethosuximide | DB00593 | e | 0 | Bi-directional transwell transport with MDCKII, MDCKII/MDR1, and MDCKII/Mdr1a cells, ATPase, and Calcein AM accumulation inhibition | [15] |
| Farnesol | CID_445070 | e | 0 | ATPase activity | [9] |
| Hydrocodone | DB00956 | e | 0 | Bi-directional transwell transport with MDCKII, MDCKII/MDR1, and MDCKII/Mdr1a cells, ATPase, and Calcein AM accumulation inhibition | [15] |
| Itraconazole | CID_55283 | e | 0 | ATPase, Calcein AM accumulation, permeability | [2] |
| Levetiracetam | DB01202 | e | 0 | Bi-directional transwell transport with LLC-PK1/MDR1 cells | [42,51] |
| Maprotiline | DB00934 | e | 0 | Calcein AM accumulation, permeability | [14] |
| Noscapine | CID_4544 | e | 0 | Calcein AM accumulation, permeability | [14] |
| Practolol | CID_4883 | e | 0 | ATPase, Calcein AM accumulation, permeability | [2] |
| Progabide | CID_5361323 | e | 0 | Calcein AM accumulation, permeability | [14] |
| Sulfamethoxazole | DB01015 | e | 0 | Bi-directional transwell transport with MDCKII and MDCKII/MDR1 cells | [20] |
| Trazodone | CID_5533 | e | 0 | Calcein AM accumulation, permeability | [14] |
| Venlafaxine | DB00285 | e | 0 | Bi-directional transwell transport with MDCKII, MDCKII/MDR1, and MDCKII/Mdr1a cells, ATPase, and Calcein AM accumulation inhibition | [15] |

**References**

1. Pauli-Magnus C, Murdter T, Godel A, Mettang T, Eichelbaum M, et al. (2001) P-glycoprotein-mediated transport of digitoxin, alpha-methyldigoxin and beta-acetyldigoxin. Naunyn Schmiedebergs Arch Pharmacol 363: 337-343.

2. Polli JW, Wring SA, Humphreys JE, Huang L, Morgan JB, et al. (2001) Rational use of in vitro P-glycoprotein assays in drug discovery. J Pharmacol Exp Ther 299: 620-628.

3. Genne P, Dimanche-Boitrel MT, Mauvernay RY, Gutierrez G, Duchamp O, et al. (1992) Cinchonine, a potent efflux inhibitor to circumvent anthracycline resistance in vivo. Cancer Res 52: 2797-2801.

4. Schwab D, Fischer H, Tabatabaei A, Poli S, Huwyler J (2003) Comparison of in vitro P-glycoprotein screening assays: recommendations for their use in drug discovery. J Med Chem 46: 1716-1725.

5. Hochman JH, Pudvah N, Qiu J, Yamazaki M, Tang C, et al. (2004) Interactions of human P-glycoprotein with simvastatin, simvastatin acid, and atorvastatin. Pharm Res 21: 1686-1691.

6. Safa AR (1993) Photoaffinity labeling of P-glycoprotein in multidrug-resistant cells. Cancer Invest 11: 46-56.

7. Ferry DR, Traunecker H, Kerr DJ (1996) Clinical trials of P-glycoprotein reversal in solid tumours. Eur J Cancer 32A: 1070-1081.

8. Yates CR, Chang C, Kearbey JD, Yasuda K, Schuetz EG, et al. (2003) Structural determinants of P-glycoprotein-mediated transport of glucocorticoids. Pharm Res 20: 1794-1803.

9. Zhang L, Sachs CW, Fine RL, Casey PJ (1994) Interaction of prenylcysteine methyl esters with the multidrug resistance transporter. J Biol Chem 269: 15973-15976.

10. Obradovic T, Dobson GG, Shingaki T, Kungu T, Hidalgo IJ (2007) Assessment of the first and second generation antihistamines brain penetration and role of P-glycoprotein. Pharm Res 24: 318-327.

11. Gosland MP, Lum BL, Sikic BI (1989) Reversal by cefoperazone of resistance to etoposide, doxorubicin, and vinblastine in multidrug resistant human sarcoma cells. Cancer Res 49: 6901-6905.

12. Akiyama S, Cornwell MM, Kuwano M, Pastan I, Gottesman MM (1988) Most drugs that reverse multidrug resistance also inhibit photoaffinity labeling of P-glycoprotein by a vinblastine analog. Mol Pharmacol 33: 144-147.

13. Kivisto KT, Zukunft J, Hofmann U, Niemi M, Rekersbrink S, et al. (2004) Characterisation of cerivastatin as a P-glycoprotein substrate: studies in P-glycoprotein-expressing cell monolayers and mdr1a/b knock-out mice. Naunyn Schmiedebergs Arch Pharmacol 370: 124-130.

14. Mahar Doan KM, Humphreys JE, Webster LO, Wring SA, Shampine LJ, et al. (2002) Passive permeability and P-glycoprotein-mediated efflux differentiate central nervous system (CNS) and non-CNS marketed drugs. J Pharmacol Exp Ther 303: 1029-1037.

15. Feng B, Mills JB, Davidson RE, Mireles RJ, Janiszewski JS, et al. (2008) In vitro P-glycoprotein assays to predict the in vivo interactions of P-glycoprotein with drugs in the central nervous system. Drug Metab Dispos 36: 268-275.

16. Kajiji S, Dreslin JA, Grizzuti K, Gros P (1994) Structurally distinct MDR modulators show specific patterns of reversal against P-glycoproteins bearing unique mutations at serine939/941. Biochemistry 33: 5041-5048.

17. Beck WT, Cirtain MC, Glover CJ, Felsted RL, Safa AR (1988) Effects of indole alkaloids on multidrug resistance and labeling of P-glycoprotein by a photoaffinity analog of vinblastine. Biochem Biophys Res Commun 153: 959-966.

18. Schinkel AH, Wagenaar E, Mol CA, van Deemter L (1996) P-glycoprotein in the blood-brain barrier of mice influences the brain penetration and pharmacological activity of many drugs. J Clin Invest 97: 2517-2524.

19. Safa AR, Agresti M, Bryk D, Tamai I (1994) N-(p-azido-3-[125I]iodophenethyl)spiperone binds to specific regions of P-glycoprotein and another multidrug binding protein, spiperophilin, in human neuroblastoma cells. Biochemistry 33: 256-265.

20. Susanto M, Benet LZ (2002) Can the enhanced renal clearance of antibiotics in cystic fibrosis patients be explained by P-glycoprotein transport? Pharm Res 19: 457-462.

21. Ford JM (1996) Experimental reversal of P-glycoprotein-mediated multidrug resistance by pharmacological chemosensitisers. Eur J Cancer 32A: 991-1001.

22. Evans DC, O'Connor D, Lake BG, Evers R, Allen C, et al. (2003) Eletriptan metabolism by human hepatic CYP450 enzymes and transport by human P-glycoprotein. Drug Metab Dispos 31: 861-869.

23. Moller M, Weiss J, Wink M (2006) Reduction of cytotoxicity of the alkaloid emetine through P-glycoprotein (MDR1/ABCB1) in human Caco-2 cells and leukemia cell lines. Planta Med 72: 1121-1126.

24. Kim WY, Benet LZ (2004) P-glycoprotein (P-gp/MDR1)-mediated efflux of sex-steroid hormones and modulation of P-gp expression in vitro. Pharm Res 21: 1284-1293.

25. Florea BI, van der Sandt IC, Schrier SM, Kooiman K, Deryckere K, et al. (2001) Evidence of P-glycoprotein mediated apical to basolateral transport of flunisolide in human broncho-tracheal epithelial cells (Calu-3). Br J Pharmacol 134: 1555-1563.

26. Dey S, Ramachandra M, Pastan I, Gottesman MM, Ambudkar SV (1997) Evidence for two nonidentical drug-interaction sites in the human P-glycoprotein. Proc Natl Acad Sci U S A 94: 10594-10599.

27. Ueda K, Okamura N, Hirai M, Tanigawara Y, Saeki T, et al. (1992) Human P-glycoprotein transports cortisol, aldosterone, and dexamethasone, but not progesterone. J Biol Chem 267: 24248-24252.

28. Huwyler J, Drewe J, Klusemann C, Fricker G (1996) Evidence for P-glycoprotein-modulated penetration of morphine-6-glucuronide into brain capillary endothelium. Br J Pharmacol 118: 1879-1885.

29. Pauli-Magnus C, Rekersbrink S, Klotz U, Fromm MF (2001) Interaction of omeprazole, lansoprazole and pantoprazole with P-glycoprotein. Naunyn Schmiedebergs Arch Pharmacol 364: 551-557.

30. Morimoto K, Nakakariya M, Shirasaka Y, Kakinuma C, Fujita T, et al. (2008) Oseltamivir (Tamiflu) efflux transport at the blood-brain barrier via P-glycoprotein. Drug Metab Dispos 36: 6-9.

31. Thimmaiah KN, Horton JK, Qian XD, Beck WT, Houghton JA, et al. (1990) Structural determinants of phenoxazine type compounds required to modulate the accumulation of vinblastine and vincristine in multidrug-resistant cell lines. Cancer Commun 2: 249-259.

32. Pohl A, Lage H, Muller P, Pomorski T, Herrmann A (2002) Transport of phosphatidylserine via MDR1 (multidrug resistance 1)P-glycoprotein in a human gastric carcinoma cell line. Biochem J 365: 259-268.

33. Siest G, Jeannesson E, Berrahmoune H, Maumus S, Marteau JB, et al. (2004) Pharmacogenomics and drug response in cardiovascular disorders. Pharmacogenomics 5: 779-802.

34. Rautio J, Humphreys JE, Webster LO, Balakrishnan A, Keogh JP, et al. (2006) In vitro p-glycoprotein inhibition assays for assessment of clinical drug interaction potential of new drug candidates: a recommendation for probe substrates. Drug Metab Dispos 34: 786-792.

35. Nare B, Prichard RK, Georges E (1994) Characterization of rhodamine 123 binding to P-glycoprotein in human multidrug-resistant cells. Mol Pharmacol 45: 1145-1152.

36. Cummins CL, Jacobsen W, Christians U, Benet LZ (2004) CYP3A4-transfected Caco-2 cells as a tool for understanding biochemical absorption barriers: studies with sirolimus and midazolam. J Pharmacol Exp Ther 308: 143-155.

37. Wu J, Furusawa S, Nakano S, Takahashi M, Chiba H, et al. (1996) Reversal of multidrug resistance by tacrolimus hydrate. Methods Find Exp Clin Pharmacol 18: 651-658.

38. Hendricks CB, Rowinsky EK, Grochow LB, Donehower RC, Kaufmann SH (1992) Effect of P-glycoprotein expression on the accumulation and cytotoxicity of topotecan (SK&F 104864), a new camptothecin analogue. Cancer Res 52: 2268-2278.

39. Kyriakopoulos M, Perez-Iglesias R, Woolley JB, Kanaan RA, Vyas NS, et al. (2009) Effect of age at onset of schizophrenia on white matter abnormalities. Br J Psychiatry 195: 346-353.

40. Ford JM, Prozialeck WC, Hait WN (1989) Structural features determining activity of phenothiazines and related drugs for inhibition of cell growth and reversal of multidrug resistance. Mol Pharmacol 35: 105-115.

41. Safa AR, Roberts S, Agresti M, Fine RL (1994) Tamoxifen aziridine, a novel affinity probe for P-glycoprotein in multidrug resistant cells. Biochem Biophys Res Commun 202: 606-612.

42. Baltes S, Gastens AM, Fedrowitz M, Potschka H, Kaever V, et al. (2007) Differences in the transport of the antiepileptic drugs phenytoin, levetiracetam and carbamazepine by human and mouse P-glycoprotein. Neuropharmacology 52: 333-346.

43. Uhr M, Grauer MT, Holsboer F (2003) Differential enhancement of antidepressant penetration into the brain in mice with abcb1ab (mdr1ab) P-glycoprotein gene disruption. Biol Psychiatry 54: 840-846.

44. Zamora JM, Pearce HL, Beck WT (1988) Physical-chemical properties shared by compounds that modulate multidrug resistance in human leukemic cells. Mol Pharmacol 33: 454-462.

45. Kan WM, Liu YT, Hsiao CL, Shieh CY, Kuo JH, et al. (2001) Effect of hydroxyzine on the transport of etoposide in rat small intestine. Anticancer Drugs 12: 267-273.

46. Kim RB, Wandel C, Leake B, Cvetkovic M, Fromm MF, et al. (1999) Interrelationship between substrates and inhibitors of human CYP3A and P-glycoprotein. Pharm Res 16: 408-414.

47. Pearce HL, Safa AR, Bach NJ, Winter MA, Cirtain MC, et al. (1989) Essential features of the P-glycoprotein pharmacophore as defined by a series of reserpine analogs that modulate multidrug resistance. Proc Natl Acad Sci U S A 86: 5128-5132.

48. Liang E, Proudfoot J, Yazdanian M (2000) Mechanisms of transport and structure-permeability relationship of sulfasalazine and its analogs in Caco-2 cell monolayers. Pharm Res 17: 1168-1174.

49. Oostendorp RL, Marchetti S, Beijnen JH, Mazzanti R, Schellens JH (2007) The effect of hydroxyurea on P-glycoprotein/BCRP-mediated transport and CYP3A metabolism of imatinib mesylate. Cancer Chemother Pharmacol 59: 855-860.

50. Liu XD, Liu GQ (2001) P glycoprotein regulated transport of glutamate at blood brain barrier. Acta Pharmacol Sin 22: 111-116.

51. Luna-Tortos C, Fedrowitz M, Loscher W (2008) Several major antiepileptic drugs are substrates for human P-glycoprotein. Neuropharmacology 55: 1364-1375.
