# Supplementary material for: Predicting P-Glycoprotein-Mediated Drug Transport Based On Support Vector Machine and Three-Dimensional Crystal Structure of P-glycoprotein
Source: PLoS One. 2011 Oct 4;6(10):e25815. doi: 10.1371/journal.pone.0025815 (PMC3186768; doi:10.1371/journal.pone.0025815)
Supplement: Table S2 — SVM prediction performance parameters of the 6 best models. (DOCX) [file pone.0025815.s002.docx]

**Table S2. SVM prediction performance parameters of the 6 best models.** ACC, SP, SE, and MCC represent accuracy, specificity, sensitivity, and the Matthews correlation coefficient, respectively.

|  |  | Training Set | | | | Test Set | | | | External Validation Set | | | |
| --- | --- | --- | --- | --- | --- | --- | --- | --- | --- | --- | --- | --- | --- |
| Model | No. of Descriptor | ACC | SP | SE | MCC | ACC | SP | SE | MCC | ACC | SP | SE | MCC |
| 1 | 48 | 75.8 | 75.3 | 79.7 | 0.5138 | 75.8 | 63.2 | 92.3 | 0.5666 | 78.1 | 76.5 | 81.3 | 0.56360 |
| 2 | 48 | 78.8 | 78.2 | 72.9 | 0.5692 | 78.8 | 100.0 | 69.6 | 0.6397 | 75.0 | 78.6 | 68.8 | 0.50395 |
| 3 | 48 | 75.8 | 76.9 | 74.6 | 0.5155 | 75.8 | 70.6 | 80.0 | 0.5203 | 78.1 | 80.0 | 75.0 | 0.56360 |
| 4 | 12 | 78.8 | 80.8 | 80.8 | 0.5709 | 78.8 | 57.1 | 88.9 | 0.5757 | 78.1 | 76.5 | 81.3 | 0.56360 |
| 5 | 6 | 78.8 | 77.9 | 80.3 | 0.5760 | 78.8 | 76.5 | 81.3 | 0.5772 | 78.1 | 80.0 | 75.0 | 0.56360 |
| 6 | 6 | 78.8 | 77.1 | 81.8 | 0.5768 | 78.8 | 80.0 | 75.0 | 0.5756 | 75.0 | 75.0 | 75.0 | 0.50000 |
