# Supplementary material for: Predicting P-Glycoprotein-Mediated Drug Transport Based On Support Vector Machine and Three-Dimensional Crystal Structure of P-glycoprotein
Source: PLoS One. 2011 Oct 4;6(10):e25815. doi: 10.1371/journal.pone.0025815 (PMC3186768; doi:10.1371/journal.pone.0025815)
Supplement: Table S3 — Prediction results in the external validation data set by the 6 best models. (DOCX) [file pone.0025815.s003.docx]

**Table S3. Prediction results in the external validation data set by the 6 best models**

TP: true positive; FP: false positive; TN: true negative; FN: false negative result

| Compound | DrugBank/Pubchem ID | experimental class | MODEL# | | | | | |
| --- | --- | --- | --- | --- | --- | --- | --- | --- |
|  |  |  | 1 | 2 | 3 | 4 | 5 | 6 |
| Actinomycin D | CID_2019 | substrate | TP | TP | TP | TP | TP | TP |
| Aldosterone | CID_5839 | substrate | TP | TP | TP | TP | TP | TP |
| Bisbenzimide | CID_1464 | substrate | TP | TP | TP | TP | TP | TP |
| Catharanthin | CID_197771 | substrate | TP | TP | TP | TP | TP | TP |
| Colchicine | DB01394 | substrate | TP | TP | TP | TP | TP | TP |
| Crixivan | CID_5362440 | substrate | TP | TP | TP | TP | TP | TP |
| Fluphenazine | CID_3372 | substrate | TP | TP | TP | TP | TP | TP |
| Imatinib | DB00619 | substrate | TP | TP | TP | TP | TP | TP |
| Methysergide | CID_9681 | substrate | TP | TP | TP | TP | TP | TP |
| Mitoxantrone | CID_4212 | substrate | TP | TP | TP | TP | TP | TP |
| Navelbine base | CID_60780 | substrate | TP | TP | TP | TP | TP | TP |
| Puromycin | DB08437 | substrate | TP | TP | TP | TP | TP | TP |
| Alprenolol | CID_2119 | non-substrate | TN | TN | TN | TN | TN | TN |
| Chlorprothixene | CID_667467 | non-substrate | TN | TN | TN | TN | TN | TN |
| Diphenhydramine | CID_3100 | non-substrate | TN | TN | TN | TN | TN | TN |
| Ethosuximide | DB00593 | non-substrate | TN | TN | TN | TN | TN | TN |
| Farnesol | CID_445070 | non-substrate | TN | TN | TN | TN | TN | TN |
| Gabrene | CID_5361323 | non-substrate | TN | TN | TN | TN | TN | TN |
| Levetiracetam | DB01202 | non-substrate | TN | TN | TN | TN | TN | TN |
| Maprotiline | DB00934 | non-substrate | TN | TN | TN | TN | TN | TN |
| Practolol | CID_4883 | non-substrate | TN | TN | TN | TN | TN | TN |
| Sulfamethoxazole | DB01015 | non-substrate | TN | TN | TN | TN | TN | TN |
| Venlafaxine | DB00285 | non-substrate | TN | TN | TN | TN | TN | TN |
| Chloroquine | CID_2719 | substrate | FN | FN | FN | FN | FN | FN |
| Estriol | DB04573 | substrate | FN | FN | FN | FN | FN | FN |
| L-Glutamic Acid | DB00142 | substrate | FN | FN | FN | FN | FN | FN |
| Doxapram | CID_3156 | non-substrate | FP | FP | FP | FP | FP | FP |
| Itraconazole | CID_55283 | non-substrate | FP | FP | FP | FP | FP | FP |
| Narcotine | CID_4544 | non-substrate | FP | FP | FP | FP | FP | FP |
| Lansoprazole | DB00448 | substrate | TP | FN | TP | FN | FN | TP |
| Hydrocodone | DB00956 | non-substrate | TN | TN | FP | TN | TN | TN |
| Trazodone | CID_5533 | non-substrate | FP | TN | FP | FP | TN | FP |
